# The Effect of CO_2_ on Resting-State Functional Connectivity: Isocapnia vs. Poikilocapnia

**DOI:** 10.3389/fphys.2021.639782

**Published:** 2021-05-13

**Authors:** Larissa McKetton, Kevin Sam, Julien Poublanc, Adrian P. Crawley, Olivia Sobczyk, Lakshmikumar Venkatraghavan, James Duffin, Joseph A. Fisher, David J. Mikulis

**Affiliations:** ^1^Division of Neuroradiology, Joint Department of Medical Imaging, University Health Network, Toronto, ON, Canada; ^2^The Russell H. Morgan Department of Radiology & Radiological Science, The John Hopkins University School of Medicine, Baltimore, MD, United States; ^3^Institute of Medical Sciences, The University of Toronto, Toronto, ON, Canada; ^4^Department of Anesthesia and Pain Management, University Health Network, Toronto, ON, Canada; ^5^Department of Physiology, The University of Toronto, Toronto, ON, Canada

**Keywords:** BOLD, end-tidal pressure of CO_2_, functional-connectivity, fMRI, resting-state

## Abstract

The normal variability in breath size and frequency results in breath-to-breath variability of end-tidal PCO_2_ (P_ET_CO_2_), the measured variable, and arterial partial pressure of carbon dioxide (PaCO_2_), the independent variable affecting cerebral blood flow (CBF). This study examines the effect of variability in PaCO_2_ on the pattern of resting-state functional MRI (rs-fMRI) connectivity. A region of interest (ROI)-to-ROI and Seed-to-Voxel first-level bivariate correlation, hemodynamic response function (hrf)-weighted analysis for measuring rs-fMRI connectivity was performed during two resting-state conditions: (a) normal breathing associated with breath-to-breath variation in PaCO_2_ (poikilocapnia), and (b) normal breathing with breath-to-breath variability of P_ET_CO_2_ dampened using sequential rebreathing (isocapnia). End-tidal PCO_2_ (P_ET_CO_2_) was used as a measurable surrogate for fluctuations of PaCO_2_. During poikilocapnia, enhanced functional connections were found between the cerebellum and inferior frontal and supramarginal gyrus (SG), visual cortex and occipital fusiform gyrus; and between the primary visual network (PVN) and the hippocampal formation. During isocapnia, these associations were not seen, rather enhanced functional connections were identified in the corticostriatal pathway between the putamen and intracalacarine cortex, supracalcarine cortex (SCC), and precuneus cortex. We conclude that vascular responses to variations in P_ET_CO_2_, account for at least some of the observed resting state synchronization of blood oxygenation level-dependent (BOLD) signals.

## Introduction

The pioneering work of Biswal et al. (1995) showed that low-frequency (<0.1 Hz) fluctuations of EPI signal intensity were temporally synchronized across the non-contiguous motor cortex during resting-state functional MRI (rs-fMRI). This finding led to the development of a stimulus-free methodology for the assessment of functional brain networks. The correlated fluctuations within brain regions are thought to be synchronized variations in neuronal activity revealing functional connectivity between brain regions. However, one of the major challenges involved in the analysis of functional connectivity stems from problems separating the blood oxygenation level-dependent (BOLD) fMRI signals of neuronal stimulation, from those resulting from changes in arterial partial pressure of carbon dioxide (PaCO_2_). PaCO_2_ is a potent vasoactive molecule, which varies breath-to-breath due to differences in tidal volume during normal breathing (Wise et al., 2004; Birn et al., 2006; Madjar et al., 2012). Wise et al. (2004) found BOLD fMRI fluctuations in disparate brain regions and variability in the middle cerebral artery (MCA) flow velocity measured by transcranial Doppler that were correlated with changes in the end-tidal PCO_2_ (P_ET_CO_2_), a non-invasive surrogate of PaCO_2_. The synchronous changes were widespread and bilaterally symmetrical, particularly in gray matter (GM) of the occipital, parietal, temporal, and cingulate cortex, and to a lesser extent in the white matter (WM) (Wise et al., 2004). P_ET_CO_2_ fluctuations occur around 0.03 Hz in GM (Wise et al., 2004). This frequency of respiratory changes overlaps with resting-state brain activity frequency fluctuations of approximately 0.1 Hz, which are typically not filtered out by physiological noise correction routines and may confound resting-state neuronal functional connectivity measures. Eliminating P_ET_CO_2_ fluctuations would dampen respiratory based synchronization leaving those of neuronal origin.

The relative contributions of neuronal and vascular factors comprising the BOLD signal during rs-fMRI are still unknown. In a pivotal study, Golestani et al. (2015) reported that spontaneous P_ET_CO_2_ fluctuations act as strong modulators of the rs-fMRI signal contributing up to 15% of the total rs-fMRI signal variance based on a multi-regression model used to estimate the voxel-wise P_ET_CO_2_ response functions. These CO_2_ fluctuations primarily affect fMRI signals in the GM, particularly in the occipital and temporal cortices in addition to subcortical structures including the precuneus, cingulate gyrus, and thalamus (Golestani et al., 2015).

Consequently, we studied BOLD signal synchronization in healthy volunteers under two conditions: (1) spontaneous breathing that is associated with normal P_ET_CO_2_ variability (poikilocapnia), and (2) P_ET_CO_2_ variability dampened by sequential gas delivery (isocapnia) (Fierstra et al., 2013; Fisher, 2016). We hypothesized that the regions showing synchronous BOLD fluctuations during poikilocapnia but not isocapnia are synchronized to P_ET_CO_2_. The synchronized regions during isocapnia therefore likely reflect neuronal resting state functional connectivity under isocapnic breathing conditions.

## Materials and Methods

### Participants and Image Acquisition

This study conformed to the standards set by the latest revision of the Declaration of Helsinki and was approved by the Institution’s Research Ethics Board. Written informed consent was obtained from all participants. Ten healthy control participants with no history of neurological disorders [age range: 22–70; eight males; age mean (SD) 42.4 (18.68)] were recruited.

All images were acquired using a 3-Tesla GE MRI scanner (Signa HDx, GE Healthcare, Milwaukee, WI, United States), using an eight-channel phased array head coil. Participants had at least 10 min at rest in the scanner before the fMRI time series acquisition. High-resolution T1-weighted 3D spoiled gradient echo sequences were acquired with the following parameters: TR = 7.88 ms, TE = 3 ms, flip angle = 12^°^, 146 slices, voxel size = 0.85 × 0.85 × 1 mm, matrix size = 256 × 256, and field of view = 22 × 22 cm. Two BOLD fMRI echoplanar images were acquired, one for each resting-state condition with the following parameters: TR = 2400 ms, TE = 30 ms, flip angle = 70^°^, 250 volumes, 41 slices, isotropic voxel size = 3.5 mm, matrix size = 64 × 64, and field of view = 24 × 24 cm.

### Control of Blood Gases During Spontaneous Breathing

The dampening of breath-to-breath P_ET_CO_2_ and the end-tidal partial pressure of O_2_ (P_ET_O_2_) was achieved using an automated gas blender that applies sequential gas delivery algorithms targeting resting P_ET_CO_2_ (RespirAct™, Thornhill Research Inc., Canada; Slessarev et al., 2007; Fierstra et al., 2013; Fisher, 2016) thereby dampening physiologically significant breath-to-breath variation in P_ET_CO_2_. Subjects breathed via a soft plastic mask sealed to their face using transparent dressing film (Tegaderm, 3M, St. Paul MN, United States) during poikilocapnia and isocapnia. For isocapnia, P_ET_CO_2_ was controlled by targeting each subject’s resting P_ET_CO_2_; average 38 mmHg (range 32–42 mmHg). P_ET_O_2_ was targeted at 100 mmHg. During the poikilocapnic resting-state scan, participants breathed room air with no targeting of P_ET_CO_2_ while P_ET_O_2_ permitting physiological fluctuations as occurs in fMRI experiments. The sequence of isocapnia and poikilocapnia was randomized. All subjects indicated that they were unable to distinguish between the two resting-state conditions.

### Data Preprocessing

Neuroimaging data were preprocessed and analyzed using SPM12 (The Wellcome Department of Cognitive Neurology, London, United Kingdom)^1^ running in Matlab v7.14 (The Mathworks Inc., United States). All functional volumes underwent slice-timing correction using sinc interpolation to temporally align the slices within each volume. Each volume was spatially realigned to the first volume acquired using a six-parameter rigid body transformation. Realignment motion parameters were set at 3 mm for translation and 1° for rotation relative to the first volume for exclusion criteria. Head movement was measured in three axes with 1.5 mm limits with all subjects meeting this criterion. High-resolution T_1_-weighted anatomical volumes were segmented into GM, WM, and cerebrospinal fluid (CSF), and were normalized to Montreal Neurological Institute (MNI) space using the normalized EPI image in SPM. Functional images were normalized into MNI space and smoothed with a spatial convolution 8 mm full-width half-maximum Gaussian kernel.

Functional connectivity measures were analyzed in Matlab using the CONN-fMRI functional connectivity toolbox v17f (Whitfield-Gabrieli and Nieto-Castanon, 2012).^2^ Data were de-noised following the anatomical CompCor approach (Behzadi et al., 2007). CompCor is advantageous in that it does not require external monitoring of physiological fluctuations (cardiac and respiratory) as compared to other noise correction routines. Anatomical CompCor factors in the signal from WM, ventricles, large vessels, and CSF to accurately model physiological fluctuations in GM areas and uses the five most significant principal components each from the WM and CSF as covariates in a general linear model (GLM) as an estimate for physiological noise (Behzadi et al., 2007). We additionally analyzed the resting-state data using CompCor and with CompCor turned off (i.e., removing WM and CSF covariates). We expected that isocapnia would mostly remove variations due to P_ET_CO_2_ fluctuations, whereas CompCor would remove other sources of noise such as cardiac pulsations, in order to see how these two methods compared.

Global signal regression was not performed in order to bypass introduction of artifactually negative correlations into resulting connectivity measures (Chai et al., 2012). Instead, a number of confounds were regressed out via CompCor to further remove unwanted BOLD signal artifactual effects that were shown to improve sensitivity, specificity, and validity for subsequent functional connectivity analyses. In addition to the CompCor covariates, realignment motion regression (12 regressors comprised of six motion regressors and six first-order temporal derivatives), and functional outliers that were detected via the ART-based identification of outlier scans for scrubbing, thresholded at 0.9 mm for framewise displacement (Power et al., 2012) were also included. As recommended, band-pass filtering was also performed with a frequency window of 0.008–0.09Hz. This preprocessing step helps increase retest reliability, while reducing the effects of low frequency drift and high frequency noise (Weissenbacher et al., 2009). Linear detrending was performed as another de-noising parameter to remove linear trends within each functional scan.

### P_ET_CO_2_ and BOLD Correlation Analysis

**Figure 1:**
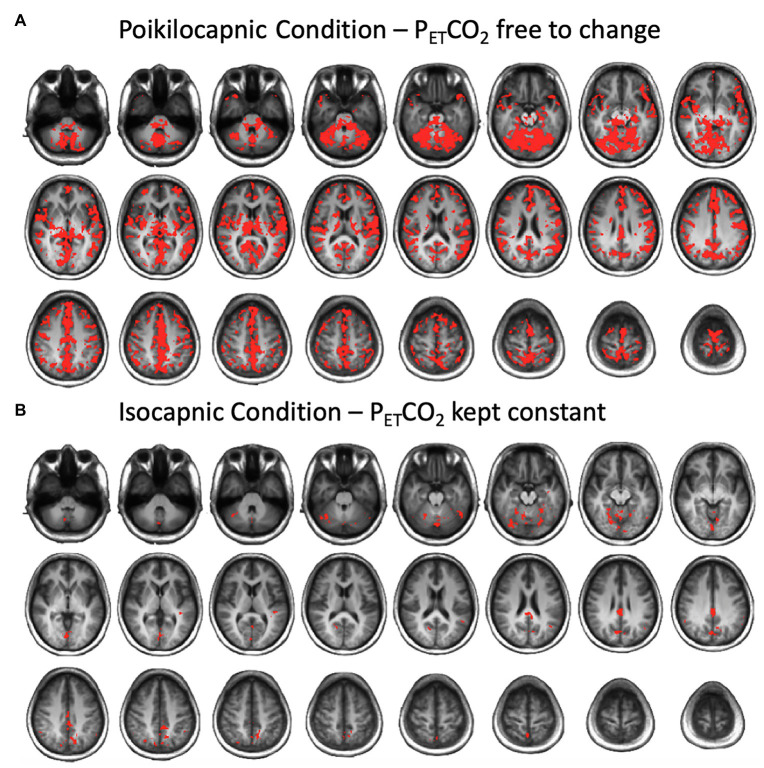
Brain average maps displaying the correlations between the blood oxygenation level-dependent (BOLD) signal and end-tidal PCO_2_ (P_ET_CO_2_) time course resting state conditions after multiple comparison correction using threshold free cluster enhancement (TFCE) in **(A)** the poikilocapnic condition, and **(B)** the isocapnic condition. Significant regions at *p* < 0.05 are shown.

End-tidal PCO_2_ was shifted to the maximum correlation with the average BOLD signal using Matlab. Correlation maps between P_ET_CO_2_ and the resting BOLD signal were computed for each resting-state condition using AFNI software (National Institutes of Health; Cox, 1996) with quadratic fitting to control for baseline and trend. Resulting images were reregistered to MNI space. After Fisher’s Z transformation of the correlation values, group analysis was computed for each group to compare correlations between the P_ET_CO_2_ time course and BOLD signal during both resting-state conditions. Threshold-Free Cluster Enhancement (TFCE) was performed on the correlations between the BOLD signal and P_ET_CO_2_ time course data. TFCE is an optimal method in enhancing cluster like structures that has been shown to provide better sensitivity than other methods throughout an extensive range of test signal shapes, signal-to-noise ratios (SNRs), and has been described in detail previously (Smith and Nichols, 2009). Permutation testing was then applied to the height of the maxima of the resulting statistic image, using the “randomize” permutation-based inference tool (Winkler et al., 2014) in FSL v.5.0.9 (FMRIB Library)^3^ that allowed for the maintenance of strong control over family-wise error (FWE). The effect of spontaneous breathing (poikilocapnia) and (isocapnia) were tested for multiple comparisons, where any significant regions at *p* < 0.05 were reported and illustrated (Figure 1).

### Connectivity Analysis

An ROI-to-ROI and Seed-to-Voxel first-level bivariate correlation, hrf-weighted analysis was conducted using a functional connectivity (weighted GLM) model for measuring resting-state connectivity.

The first exploratory whole-brain ROI-to-ROI functional connectivity analysis was performed to examine an unbiased investigation of two types of ROIs within CONN tool. This consisted of a group of 132 ROIs and the second group of 31 ROIs across eight networks that were commonly assigned across subjects. The 132 ROI group was based on the FSL Harvard-Oxford Atlas including 91 cortical, 15 subcortical ROIs [developed at the Center for Morphometric Analysis (CMA), and distributed with the FMRIB Library in FSL], and 26 cerebellar parcellations from the Automated Anatomical Labeling (AAL) Atlas (Tzourio-Mazoyer et al., 2002). The 31 ROI group was derived across eight networks e.g., default mode network (DMN); medial prefrontal cortex (MPFC); posterior cingulate cortex (PCC); right lateral parietal (RLP); and left lateral parietal (LLP) cortex areas. For each ROI, the mean BOLD signal time series was extracted within the ROI voxels, and bivariate correlation coefficients were computed for each pair of ROIs that were Fisher *z*-transformed. For second-level analysis, a two-sided within-subject paired *t*-test for all subjects between-conditions contrast of isocapnia vs. poikilocapnia was applied using the false discovery rate (FDR) correction for multiple comparisons *p* < 0.05. Only significant ROI-to-ROI results are illustrated and reported.

The second hypothesis driven seed-to-voxel analysis was performed using 10 mm spheres positioned in ROIs that have been associated with respiration and rs-fMRI that included the cerebellum, insular/orbitofrontal/cingulate/precuneus/prefrontal cortex, putamen, caudate, superior temporal/supramarginal gyri (Birn et al., 2006; Chang and Glover, 2009), and visual cortex (Madjar et al., 2012). The mean time series were extracted in each seed region by averaging across all voxels for each participant. Bivariate correlation coefficients were computed between the seed time-course and with every other voxel in the brain. The subsequent whole-brain correlation maps were Fisher *z*-transformed producing *z*-value maps of voxel-wise functional connectivity for each seed ROI. For second-level analysis, a two-sided within-subject paired *t*-test for all subjects between-conditions contrast of isocapnia vs. poikilocapnia was applied for voxel-wise statistics throughout the whole brain at an uncorrected level (*p* < 0.001) before FWE correction was applied at the cluster level (*p* < 0.05) for multiple comparisons. Only significant seed-to-voxel results are illustrated and reported.

## Results

### Respiratory Data Results

**Figure 2:**
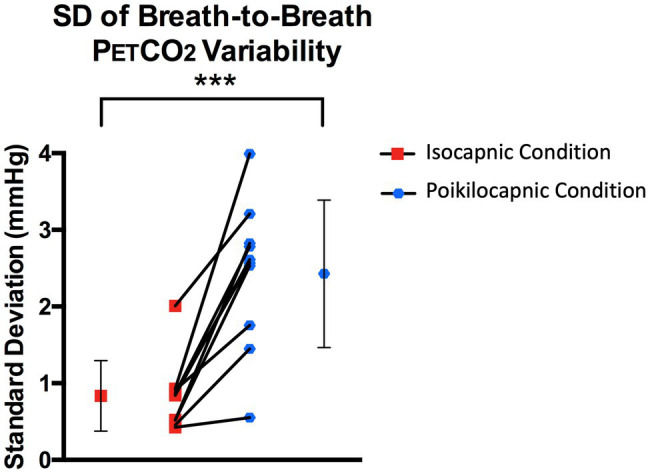
Respiratory data for both resting-state conditions. SD of breath-to-breath P_ET_CO_2_ variability in the isocapnic spontaneous breathing condition (when P_ET_CO_2_ was kept constant) denoted with red squares, and poikilocapnic spontaneous breathing condition (when P_ET_CO_2_ was free to change) denoted with blue circles. Connecting lines in the middle of the graph associate each individual subject’s condition (the mean SD of breath-to-breath variability for the isocapnic and poikilocapnic conditions). The symbol and error bars beside each line graph side denotes the mean and SD of all 10 subjects for each condition. *** denotes *p* < 0.001.

The SD of the breath-to-breath spontaneous P_ET_CO_2_ variability was significantly lower in the clamped condition 0.84 (0.46) mmHg compared to the unclamped condition 2.47 (0.96) mmHg [mean of variability (SD)], *T*(9) = 6.06, *p* = 0.0002 (Figure 2). The mean (SD) P_ET_CO_2_ was higher in the isocapnic condition 37.53 (3.04) mmHg compared to the poikilocapnic condition 33.81 (3.3) mmHg, *T*(9) = 8.38, *p* < 0.0001. This is very unlikely to be of physiologic significance for the following reason. In the isocapnic condition, the P_ET_CO_2_ is equal to the PaCO_2_ (Ito et al., 2008; Fisher et al., 2016). In healthy people P_ET_CO_2_ is typically 2–4 mmHg less than PaCO_2_ due to a small volume of physiological alveolar deadspace (Ito et al., 2008).

### Functional Connectivity Results

**Figure 3:**
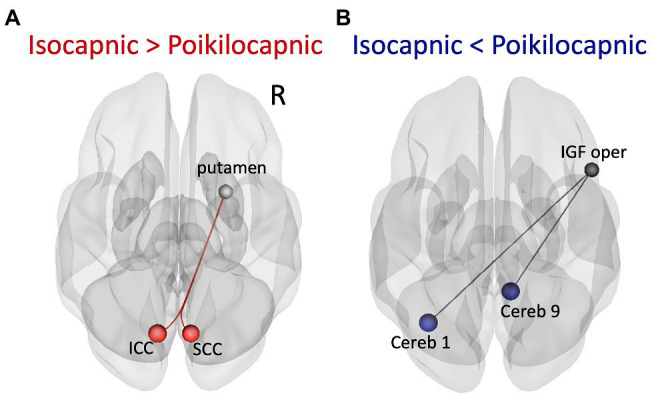
ROI-to-ROI functional connectivity results. **(A)** Superior axial view of significantly increased functional connectivity in the isocapnic condition (when P_ET_CO_2_ was kept constant) compared to the poikilocapnic condition (when P_ET_CO_2_ was free to change) between the right putamen ROI seed and left intracalcarine cortex (ICC) and right supracalcarine cortex (SCC). **(B)** Superior axial view of significantly increased functional connectivity in the poikilocapnic condition compared to the isocapnic condition between the right opercular part of the inferior frontal gyrus (IFG) seed and left Cerebellum region 1 and right Cerebellum region 9.

Exploratory whole-brain functional connectivity assessment within a whole-brain parcellated network of 132 ROIs and 42 networks revealed significant differences between the two resting state conditions. The controlled breathing isocapnic condition had enhanced functional connectivity between the right putamen with the left intracalcarine cortex (ICC) and the right putamen with the right supracalcarine cortex (SCC) (Figure 3A; Table 1). The poikilocapnic spontaneous breathing condition had enhanced functional connectivity between the right opercular part of the inferior frontal gyrus (IFG) with the left Cerebellar area 1 and the right opercular part of the IFG with the right Cerebellar area 9 (Figure 3B; Table 1). There were no differences between turning CompCor on or off for ROI-to-ROI results.

**Table 1:** ROI-to-ROI functional connectivity results within a whole-brain parcellations of 174 ROIs.

ROI 1	ROI 2	T Score	Beta	p-unc	p-FDR
*Contrast: Isocapnia* > *Poikilocapnia* Figure 5A (*red*)					
Right putamen	Left intracalcarine cortex	5.93	0.11	0.0002	0.0307
	Right supracalcarine cortex	5.51	0.15	0.0004	0.0307
Left intracalarine cortex	Right putamen	5.93	0.11	0.0002	0.0360
*Contrast: Isocapnia* > *Poikilocapnia* Figure 5B (*blue*)					
Right inferior frontal gyrus	Left cerebellum crus 1	5.84	0.15	0.0002	0.0256
	Right cerebellum 9	5.65	0.13	0.0003	0.0256
Left cerebellum crus 1	Right inferior frontal gyrus	5.84	0.15	0.0002	0.0407

Significant connections at a *p* < 0.05 false discovery rate (FDR) corrected threshold are reported.

**Figure 4:**
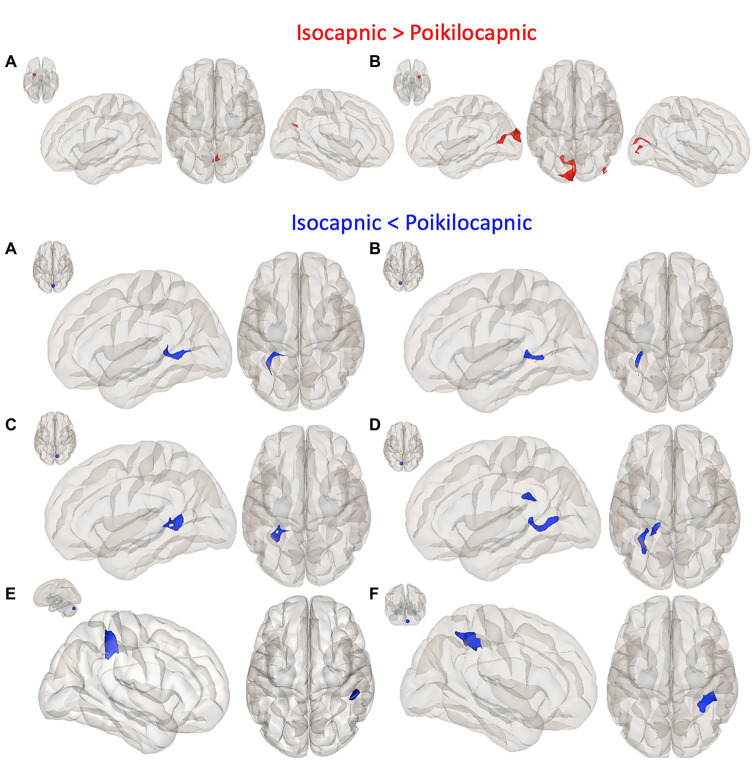
Seed-to-voxel functional connectivity results. The first panel shows greater connectivity in the isocapnic condition (red) compared to the poikilocapnic condition; seed regions (red 10 mm ROIs) are depicted by the small axial brain images (top left), and red cluster regions shown in left sagittal, superior axial, and right sagittal views. **(A)** Left putamen seed, precuneus cortex cluster; **(B)** Right putamen seed, left occipital pole and right lateral occipital cortex clusters. Panels 2–4 show greater connectivity in the poikilocapnic condition (blue) compared to the isocapnic condition; seed regions (blue 10 mm ROIs), and blue cluster regions in the left sagittal and superior axial view. **(A)** primary visual network (PVN) seed, hippocampal formation cluster; **(B)** ICC seed, Hippocampus (Hi) cluster; **(C)** left SCC seed, Hi cluster; **(D)** right SCC seed, Hi/lingual gyrus cluster; **(E)** posterior cerebellar seed; supramarginal gyrus (SG) cluster; **(F)** Cerebellum region 9 seed; superior parietal lobule (SPL) cluster. Only significant family-wise error (FWE) corrected *p* < 0.05 are reported.

In addition, a hypothesis-based seed-to-voxel analysis for contrasts comparing isocapnic to poikilocapnic conditions was calculated using second-level GLM analyses. Reported clusters were thresholded at *p* < 0.05, FWE-corrected, T maxima = 4.78. Enhanced functional connectivity in the isocapnic condition was found between the left putamen seed and a cluster in the precuneus cortex as well as the right putamen seed with a cluster in the left occipital pole and cluster in the inferior division of the right lateral occipital cortex (Figure 4; Table 2). Enhanced functional connectivity in the poikilocapnic condition was found between the primary visual network (PVN) seed and the left hippocampal formation; the left ICC and right SCC seeds with clusters in the left hippocampus; the left SCC seed with clusters in the left hippocampus/lingual gyrus, and callosal body; the posterior cerebellar network (PCN) seed with a cluster in the right supramarginal gyrus (SG); and the cerebellum region 9 seed with the a cluster in the right superior parietal lobule (SPL) (Figure 4; Table 2).

**Table 2:** Seed-to-voxel results, brain regions showing altered functional connectivity between seed and corresponding clusters within the two different resting state condition in controls comparing with CompCor and without CompCor.

	Seed	Cluster	MNI	MNI	*K*	*K*	*Beta*	*Beta*	*T*	*T*	*P*	*P*
			*CompCor*	-*CompCor*	*CompCor*	-*CompCor*	*CompCor*	-*CompCor*	*CompCor*	-*CompCor*	*CompCor*	-*CompCor*
*Isocapnia* > *Poikilocapnia* (Figure 3, *red*)												
(A)	L putamen	Precuneus cortex	−02 -72 +28	none	107	none	0.13	none	7.19	none	0.0227	none
(B)	R putamen	L occipital pole	+00–92 +16	+00–94 +14	550	518	0.14	0.14	9.88	9.72	< 0.00001	< 0.00001
		R iLOC	+46–84 +08	+46–84 +08	83	78	0.19	0.20	7.17	7.75	0.034	0.047
*Poikilocapnia* > *Isocapnia* (Figure 3, *blue*)												
(A)	PVN 2, −79, 12	L Hippocampus	−28 -40 +02	−28 -40 +02	169	154	0.18	0.18	12.66	11.96	0.0015	0.027
(B)	L ICC	L Hippocampus	−28 -40 +00	−28 -40 +00	136	134	0.19	0.18	12.26	12.05	0.0051	0.0056
(C)	R SCC	L Hippocampus	−28 -54 +10	−28 -54 +10	191	181	0.16	0.16	13.85	13.34	0.0007	0.0009
(D)	L SCC	L Hippocampus	−30 -66 +04	−32 -62 +04	233	124	0.18	0.19	11.85	11.96	0.0011	0.0098
		L Callosal Body	−18 -36 +24	none	121	none	0.15	none	11.11	none	0.0394	none
(E)	PCN 0, −79, −32	R SG	+54–36 +60	+50–32 +44	272	255	0.20	0.19	9.01	8.81	0.00003	0.00006
(F)	R Cerebellum 9	R SPL	+32–52 +52	+32–52 +52	397	379	0.16	0.16	8.80	8.91	< 0.00001	< 0.00001

Cluster location, Montreal Neurological Institute (MNI) peak cluster co-ordinates (x,y,z) and are shown in (mm), k = number of contiguous voxels in cluster (voxel size = 2 mm^3^); CompCor = using CompCor, CompCor = without CompCor; Beta = effect sizes, which represent the magnitude of connectivity (Fisher-transformed correlation coefficients) and shows the difference in means of groups (isocapnic and poikilocapnic); T = test statistic (two-sided), p = family-wise error (FWE) corrected cluster-level significance set at *p* < 0.05; L, left; R, right; PVN, primary visual network; iLOC, Lateral Occipital Cortex, inferior division. ICC, intracalcarine cortex; SCC, supracalcarine cortex; PCN, posterior cerebellar network; SG, supramarginal gyrus – posterior division; SPL, superior parietal lobule.

**Figure 5:**
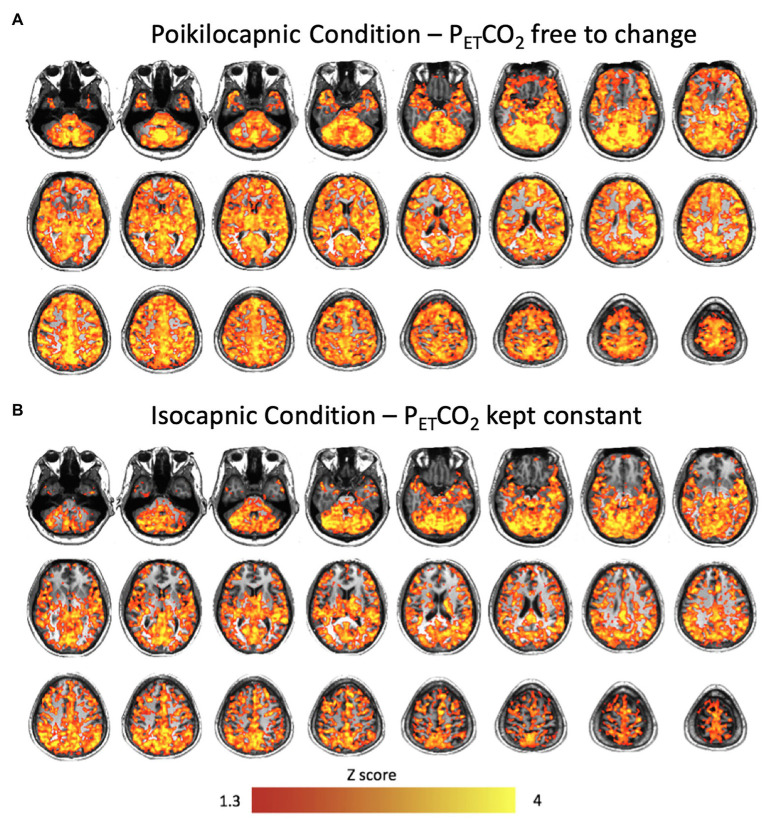
Brain average maps displaying the correlations between the BOLD signal and P_ET_CO_2_ time course during resting states in **(A)** the poikilocapnic condition, and **(B)** the isocapnic condition.

Correlation maps between the P_ET_CO_2_ and the resting BOLD revealed stronger correlations in the poikilocapnic resting state condition, where P_ET_CO_2_ was allowed to vary spontaneously as compared with the isocapnic P_ET_CO_2_ resting state condition. These regions were found predominantly in cerebellar, frontal, and occipital regions (Figure 5). In addition, statistical testing using TFCE for multiple comparison correction *p* < 0.05 was applied, where significant regions of correlations between P_ET_CO_2_ with resting BOLD are shown in red (Figure 1). Spontaneous breathing during the poikilocapnic condition showed significantly more P_ET_CO_2_ and BOLD correlations in GM cerebellar, frontal, parietal, temporal, and occipital regions (Figure 1A) compared to the isocapnic resting state condition (Figure 1B). There were minor differences when using CompCor compared without CompCor in resting state seed-to-voxel analyses.

## Discussion

### Main Findings

To the best of our knowledge, this study is the first to compare functional connectivity between poikilocapnic and isocapnic resting-state conditions using whole-brain ROI-to-ROI and seed-to-voxel analysis. The main findings of this study are that implementing isocapnia reveals neuronal functional connectivity within: (1) the corticostriatal pathway between the right putamen and ICC as well as the SCC; and (2) the left putamen and the precuneus cortex. These results are similar to those of Madjar et al. (2012) who found enhanced connectivity between the PCC and occipital regions when P_ET_CO_2_ was controlled during the resting-state acquisition. Although Madjar et al. (2012) only focused on the PCC as their seed of interest, our findings reveal other resting-state networks that were induced by respiratory changes in PCO_2_.

### PaCO_2_-Induced Synchrony of BOLD Signal in Vasoactive Tissue

Frontal, temporal, and occipital GM and cerebellum are characterized by large BOLD signal changes in response to a CO_2_ challenge (Rostrup et al., 2000; Wise et al., 2004) and therefore may also show the greatest synchronization of BOLD signals to changes in PaCO_2_ and hence to each other during poikilocapnia. Functional connections were found between the cerebellum and the IFG and SPL; the cerebellar network with the SG; the visual cortex with the occipital fusiform gyrus; the PVN with the hippocampal formation; and the ICC/SCC with the hippocampus. That these regions are synchronized by P_ET_CO_2_ fluctuation is shown by loss of synchronization with isocapnia (Figures 1, 5). These findings are consistent with those of Bright et al. (2020) who reported a significant relationship between the more vascularized visual networks during a working memory task under poikilocapnia that was not observed during isocapnia. This finding also supports the notion of a vascular driver for this functional network system as the negative BOLD signal changes were time-locked to the working memory task, whereas visual activation during the working memory task resulted in positive BOLD signal changes.

In addition, a previous study investigating the effects of speech tasks reported decreased CO_2_ pressure in the blood (hypocapnia), mainly accounting for the measured changes in oxygenation and cerebral hemodynamics. Their findings suggest the need to monitor P_ET_CO_2_ during speech tasks as PaCO_2_ is still underestimated and a potential confounder in functional brain studies (Scholkmann et al., 2013).

### Unmasking Neuronal Synchronization With Isocapnia

The percentage of BOLD signal change per increment in P_ET_CO_2_ is lowest in the striatum and thalamus (Posse et al., 1999; Wise et al., 2004), and the basal ganglia (Kastrup et al., 1999). Increased synchronization of the right putamen with the ICC during isocapnia may therefore be explained by withdrawal of confounding vasoactive stimulation, leaving undistorted neuronal synchronization. Thus, vascular responses to changes in P_ET_CO_2_ that cease with isocapnia are most likely added noise in resting-state functional connectivity analyses and therefore limit sensitivity.

### Isocapnia vs. CompCor Noise Correction

We compared resting-state analyses with and without CompCor noise correction. We found that the significant clusters from seed-to-voxel analysis were slightly larger in volume using CompCor. These results may be indicative of how isocapnic and CompCor operations remove correlated components. We expected isocapnia to mostly remove variations due to P_ET_CO_2_ fluctuations, whereas CompCor would remove other sources of noise including, for example, those due to cardiac pulsations. Tellingly, isocapnic data were unaffected by application of CompCor. It may be that isocapnia predominantly removes artifactual correlations between highly vasoactive regions, while CompCor predominantly removes artifacts from different spatial locations, revealing when they are actually operating independently. Such post processing should reduce false positives and true positives, whereas isocapnia should not reduce true positives. With control fluctuations of PaCO_2_, CompCor may be used to account for respiratory motion and cardiac pulsations, which persist with isocapnia.

### Stimulus Free Cerebrovascular Reactivity From Poikilocapnic P_ET_CO_2_ Variability

It would seem reasonable to regress out the variation due to breath-to-breath variability in P_ET_CO_2_ and generate cerebrovascular reactivity data expressed as Δ BOLD/Δ P_ET_CO_2_. Unfortunately, the general correlation between PaCO_2_ and P_ET_CO_2_ breaks down at the scale of small breath-to-breath P_ET_CO_2_ fluctuations. A small reduction in tidal volume, for example, may result in an incomplete sampling of alveolar gas at the sensor resulting in an erroneously low P_ET_CO_2_. However, the same breath also results in reduced alveolar ventilation causing an increase in PaCO_2_. This breath-by-breath divergence of PaCO_2_ from P_ET_CO_2_ confounds calculations of CVR from changes in P_ET_CO_2_, while breathing at rest. Moreover, Golestani and Chen (2020) sought to quantify the difference between rs-fMRI measures during isocapnia, poikilocapnia, and poikilocapnia after P_ET_CO_2_ correction using a CO_2_ response function (HRF_CO2_) (Golestani and Chen, 2020). Their findings revealed that clamping P_ET_CO_2_ during isocapnia did not significantly affect respiratory volume variability and cardiac-rate variation, but significantly reduced functional connectivity as calculated with voxel seeds and reduced inter-subject variability in functional connectivity. In addition, applying the P_ET_CO_2_ correction during poikilocapnia showed to increase inter-subject variability in functional connectivity (Golestani and Chen, 2020).

### Study Limitations

The small sample size may have limited our ability to detect additional regions masked by respiratory effects. However, that we have found a consistent difference in synchronization of specific brain territories with and without isocapnia despite the inter-subject variability and our small sample size suggests that the effect size of poikilocapnia is large.

Changes in perfusion pressure can affect cerebral blood flow (CBF) during hypercapnia (Aaslid et al., 1989; Perry et al., 2014). An increase in mean arterial pressure (MAP) can alter CBF in the absence of cerebral micro-vessel vasodilation (Claassen et al., 2007). The importance of accounting for MAP would help solve for the magnitude of cerebral vasodilatory response, which is important for adequately matching cerebral perfusion with neuronal metabolic demand. However, MAP cannot be measured breath-to-breath with a non-invasive blood pressure cuff, which would confound the matching of blood pressure to breath-to-breath changes in P_ET_CO_2_ and BOLD signal. In our study, we did not measure MAP. A common relationship in previous studies showed elevated MAP responses to hypercapnia in older adults compared to young adults (Claassen et al., 2007; Coverdale et al., 2017; Miller et al., 2018). However, in our studies hypercapnia was not applied and there was no reason to suspect synchrony of blood pressure with respiration. Additionally, there may be other mechanisms hampering rs-fMRI brain activity arising from other non-neuronal sources such as contributions from sympathetic vascular innervation, which may affect the fMRI signal through its connection with CBF regulation (Özbay et al., 2019) that warrant further investigation. A theoretical possibility is that eliminating the breath-to-breath variability of arterial PCO_2_ would affect the intrinsic connectivity pattern. However, the actual finding in this paper remains that the pattern seen with poikilocapnia includes false synchrony due to vasoactivity. As such, reports of connectivity data collected under poikilocapnia would need to address this issue.

### Summary

We found that isocapnia decreases or ceases the synchronization in some brain regions previously considered part of the connectome. This suggests that fluctuating P_ET_CO_2_, during the acquisition of resting-state functional data generate CO_2_-synchronized changes that would be considered false positive results for neuronal based connectivity. This artifact is particularly prominent in regions that are highly vascularized and have high vasoactive responses to PaCO_2_ such as the visual, cerebellar, and frontal regions. In those, and possibly other regions, the magnitude of the BOLD signal changes resulting from vasoactive stimulation may overwhelm those due to resting state neurovascular coupling, such as in the putamen and calcarine cortex, resulting in false negative connectivity readings in those regions. The findings indicate that resting state acquisitions would benefit from isocapnia in identifying neuronal resting state functional connectivity.

### Conclusion

The important finding in this paper remains that the pattern seen with poikilocapnia includes false synchrony due to vasoactivity. Armed with this, the ability to assess how other conditions or diseases alter DMN activity can be more accurately identified and quantitated. This has the added benefit of reducing the sample size in studies trying to determine how experimental conditions, drugs, or diseases influence activity in the DMN.

## Data Availability Statement

The raw data supporting the conclusions of this article will be made available by the authors, without undue reservation.

## Ethics Statement

The studies involving human participants were reviewed and approved by the Research Ethics Board of the University Health Network and conformed to the standards set by the latest revision of the Declaration of Helsinki. The participants provided their written informed consent to participate in this study.

## Author Contributions

LM, KS, AC, JF, and DM designed the study. LM, KS, OS, and JP collected the data. LM analyzed the data and wrote the manuscript. LM, JP, AC, OS, JF, and DM interpreted the data for the work. LM, KS, JP, AC, OS, LV, JD, JF, and DM contributed to the manuscript revision and reviewed and approved the final submission. All authors contributed to the article and approved the submitted version.

### Conflict of Interest

JF and DM are among the developers of the RespirAct™ for MRI studies at the University Health Network, part of the University of Toronto. Thornhill Research Inc. (TRI) is a for-profit biomedical manufacturing company that was spun off from UHN. It assembles the RespirAct™, on a non-profit basis to enable MRI research at UHN and around the world. JF receives income for work done for TRI and DM holds a minor equity position in TRI.

The remaining authors declare that the research was conducted in the absence of any commercial or financial relationships that could be construed as a potential conflict of interest.

## Footnotes

^1^http://www.fil.ion.ucl.ac.uk/spm/software/spm12/

^2^http://www.nitrc.org/projects/conn

^3^https://fsl.fmrib.ox.ac.uk/fsl/fslwiki/
